# Discovery of a CLN7 model of Batten disease in non-human primates

**DOI:** 10.1016/j.nbd.2018.07.013

**Published:** 2018-07-23

**Authors:** Jodi L. McBride, Martha Neuringer, Betsy Ferguson, Steven G. Kohama, Ian J. Tagge, Robert C. Zweig, Laurie M. Renner, Trevor J. McGill, Jonathan Stoddard, Samuel Peterson, Weiping Su, Larry S. Sherman, Jacqueline S. Domire, Rebecca M. Ducore, Lois M. Colgin, Anne D. Lewis

**Affiliations:** a Division of Neuroscience, Oregon National Primate Research Center, Beaverton, OR, United States; b Department of Behavioral Neuroscience, Oregon Health & Science University, Portland, OR, United States; c Division of Genetics, Oregon National Primate Research Center, Beaverton, OR, United States; d Department of Ophthalmology, Casey Eye Institute, Oregon Health & Science University, Portland, OR, United States; e Molecular and Medical Genetics, Oregon Health and Science University, Portland, OR, United States; f Advanced Imaging Research Center, Oregon Health and Science University, Portland, OR, United States; g Division of Comparative Medicine, Oregon National Primate Research Center, Beaverton, OR, United States; h Department of Cell, Developmental and Cancer Biology, Oregon Health & Science University, Portland, OR, United States

**Keywords:** CLN7, MFSD8, Late infantile neuronal ceroid lipofuscinosis, Batten disease, Non-human primate, Neurodegeneration, Retinal degeneration, Lysosomal storage disease, Japanese macaque, Large animal model

## Abstract

We have identified a natural Japanese macaque model of the childhood neurodegenerative disorder neuronal ceroid lipofuscinosis, commonly known as Batten Disease, caused by a homozygous frameshift mutation in the *CLN7* gene (*CLN7^−/−^*). Affected macaques display progressive neurological deficits including visual impairment, tremor, incoordination, ataxia and impaired balance. Imaging, functional and pathological studies revealed that *CLN7^−/−^* macaques have reduced retinal thickness and retinal function early in disease, followed by profound cerebral and cerebellar atrophy that progresses over a five to six-year disease course. Histological analyses showed an accumulation of cerebral, cerebellar and cardiac storage material as well as degeneration of neurons, white matter fragmentation and reactive gliosis throughout the brain of affected animals. This novel *CLN7^−/−^* macaque model recapitulates key behavioral and neuropathological features of human Batten Disease and provides novel insights into the pathophysiology linked to *CLN7* mutations. These animals will be invaluable for evaluating promising therapeutic strategies for this devastating disease.

## 1. Introduction

The neuronal ceroid lipofuscinoses (NCLs) are a group of heterogenic, fatal childhood neurodegenerative diseases caused by mutations in 13 different genes described to date (CLN1-CLN14; the mutation for CLN9 has yet to be identified) (Kousi et al, 2012). Today the family of NCLs are collectively referred to as Batten disease (BD), although historically the name BD was only used to describe the CLN3 variant originally characterized by Frederick Batten in 1903 (Batten, 1903). The NCLs are caused by a wide range of recessive mutations within genes encoding soluble lysosomal enzymes, integral lysosomal transmembrane proteins, endoplasmic reticulum membrane proteins and cytosolic proteins that associate with vesicular membranes (Carcel-Trullols et al., 2015). Diagnostically, the NCLs have been categorized by age of symptom onset, including infantile forms with age of onset between 0 and 2 years of age, late infantile forms with symptom onset bwtween 2–4 years of age, juvenile forms with age of onset between 5 and 10 years of age and the very rare adult onset forms with onset around 30 years of age or older (Carcel-Trullols et al., 2015).

Despite genetic heterogeneity, the constellation of neuropathological and behavioral manifestations of the NCLs are surprisingly similar, characterized by the accumulation of abundant intracytoplasmic, autofluorescent lipopigment throughout the central nervous system (CNS), neuronal degeneration and associated neuroinflammation (Anderson et al., 2013; Cooper et al., 2015; Palmer et al., 2013; Radke et al., 2015). Regional brain atrophy, storage material deposition and neuronal loss are particularly severe in the cerebellum, cerebral cortex and retina, but has also been noted in some forms of NCL in the thalamus, hippocampus and brainstem (Anderson et al., 2013; Autti et al., 2007; Cooper et al., 2015; Palmer et al., 2013; Radke et al., 2015; Tyynela et al., 2004). Peripheral organ dysfunction and storage material accumulation has also been documented outside of the CNS, including in the heart (Anderson et al., 2013; Ostergaard et al., 2011). Clinically, patients typically present first with visual deficits that ultimately progress to blindness, as well as tremors, myoclonic jerks and seizures (Kousi et al., 2012; Williams and Mole, 2012). Behavioral and speech regression is often noted by parents and is accompanied by progressive motor decline, especially ataxia and incoordination, and eventual dementia (Kousi et al., 2012; Williams and Mole, 2012). The NCLs collectively affect an estimated 1:25,000 people in the United States alone, making them one of the most prevalent childhood neurodegenerative disorders (www.ninds.gov). The NCLs are fatal and treatments for the NCLs remain largely palliative, although an enzyme replacement strategy delivering cerliponase alpha (Brineura™) to treat the motor decline associated with the CLN2 form of NCL was recently approved by the Food and Drug Administration in April of 2017.

The CLN7 form of NCL is caused by an autosomal recessive mutation in the *CLN7* gene (also known as the major facilitator superfamily domain-containing protein 8 gene, *MFSD8*; OMIM #610951). *CLN7* encodes the 518-amino acid integral lysosomal transmembrane protein, CLN7 (Aiello et al., 2009; Kousi et al., 2009; Siintola et al., 2007). To date, over 30 different human *CLN7* gene mutations have been identified, each conferring missense, nonsense or deletion/frameshift mutations, rendering the CLN7 protein severely dysfunctional or nonfunctional (Kousi et al., 2012; Sharifi et al., 2010; Siintola et al., 2007). The CLN7 protein includes twelve membrane spanning domains with cytosolic N-and C-terminal domains and, while the exact function of CLN7 remains elusive, as a member of the major facilitator superfamily of active transport proteins it is posited to transport solutes across the lysosomal membrane to aid in the digestion and clearance of cellular biomolecules (Sharifi et al., 2010; Siintola et al., 2007). Studies in rat brain show that CLN7 predominantly localizes to lysosomes and late endosomes in neurons, while expression in glia is significantly lower (Sharifi et al., 2010). The *CLN7* mutation leads to an accumulation of cellular glyco- and lipoproteins, ultimately resulting in cellular degeneration. Mutations in *CLN7* lead to the late infantile onset form of NCL, and similar to the other late infantile NCLs, the CLN7 form of BD is characterized by visual decline, speech delay, myoclonic seizures, developmental regression, incoordination, ataxia and an early death, with age of onset typically between age 2–6 (Aiello et al., 2009; Anderson et al., 2013; Craiu et al., 2015; Kousi et al., 2009; Sharifi et al., 2010; Siintola et al., 2007). MRI and CT scans from affected patients indicate cerebral and cerebellar atrophy on T1-weighted images and often an increased T2-signal in white matter (Aiello et al., 2009). Brains evaluated at autopsy show a dramatic loss of neurons in both granule and Purkinje cell layers of the cerebellum, cerebral cortical neurons (in most layers, with layer V being the most affected) as well as pyramidal neurons in the cornu ammonis (CA) regions of the hippocampus, particularly in the CA2 sub-region (Sharifi et al., 2010). Affected brain regions also contain robust accumulation of autofluorescent ceroid lipofuscin and exhibit reactive gliosis (both astrogliosis and microgliosis) (Sharifi et al., 2010). Storage accumulation has also been detected in the thalamus, basal ganglia and brainstem, without appreciable cell loss (Sharifi et al., 2010). Retinas from human CLN7 patients also exhibit a marked degree of degeneration and storage accumulation in all layers (Khan et al., 2017; Sharifi et al., 2010).

Genetic rodent models of several of the NCLs, in addition to a number of spontaneous large animal models in dogs, sheep and cattle, have been created/characterized to enable better understanding of disease pathogenesis and for the evaluation of promising therapeutics for these diseases (Shacka, 2012). A small number of partial and full knock-out mouse CLN7 models have recently been created that replicate some of the key disease features seen in human patients (Brandenstein et al., 2016; Damme et al., 2014; Jankowiak et al., 2016). Additionally, a single case report of a Chinese Crested Dog and two small cohorts of Chihuahuas that bear *CLN7* mutations have recently been characterized and show behavioral decline with accompanying accumulation of storage material, cell loss and gliosis (Ashwini et al., 2016; Faller et al., 2016; Guo et al., 2015).

In the current study, we present the first non-human primate model of any of the NCLs described to date. We have identified a cohort of ten consanguineous Japanese macaques at the Oregon National Primate Research Center (ONPRC) bearing a haplotype that includes a frameshift mutation within exon 8 of the *CLN7* gene *(CLN7^−/−^)*). We utilized a multifaceted approach that includes behavioral, genetic, imaging and neuropathological analyses to characterize this novel, spontaneous macaque model of the CLN7 variant of BD that recapitulates several of the cardinal clinical and neuropathological features of this devastating and fatal childhood disease.

## 2. Materials and methods

### 2.1. Animals

Male and female Japanese macaques (*Macaca fuscata*), age 1–25, were included in the current study. Macaques were housed in either outdoor corrals, in sheltered small-group housing or indoors in individual cages at the ONPRC. Indoor-housed animals were pair-housed and maintained on a 12-h on/12-h off lighting schedule. All animals on study had *ad libitum* access to food and water. Animal care and procedures were conducted in accordance with all federal regulations and the guidelines established by the National Institutes of Health Guide and the Institutional Animal Care and Use Committee at the ONPRC. The ONPRC is accredited by the Association for Assessment and Accreditation of Laboratory Animal Care, International.

### 2.2. Clinical observations of behavioral phenotypes

Clinical observations were conducted by at least two independent ONPRC veterinarians as well as a principle investigator with expertise in non-human primate locomotor behavior. Clinical signs were first observed while animals were in outdoor corrals or sheltered housing units. Once behavioral dysfunction was observed, animals were brought indoors to maintain their safety and for more detailed clinical observations in either larger group housing pens or in individual home cages.

### 2.3. Pedigree analysis and genetic sequencing

Archived genomic DNA samples, extracted from blood or liver tissue, were obtained from the ONPRC NHP Biobank. Whole exome libraries were prepared for nine individual Japanese macaques (3 affected, 3 parents of affected and 3 unrelated controls) using the Agilent Human Exome V6 + UTR kit following manufacturer's recommendations.). Libraries were sequenced on an Illumina HiSeq 3000 at the Oregon State University Center for Genomic Research and Biocomputing (CGRB), generating 150 bp paired end reads. The rhesus macaque genome MacaM was used for sequence alignment and variant identification (Zimin et al., 2014). Sequences were aligned with BWA-MEM (Li, 2013) and the Genome Analysis Toolkit (GATK (McKenna et al., 2010)) was used for base quality recalibration, INDEL realignment, variant discovery and genotype calling using hard filtering according to GATK Best Practices (DePristo et al., 2011; Van der Auwera et al., 2013). SnpEff was used for variant annotation and effect prediction (Cingolani et al., 2012). Genotype analysis using kin status, based on molecularly validated parentage relationships, was used to identify candidate recessive, pathogenic variants that were consistent with disease and carrier status. To validate candidate variants, target regions were PCR amplified using flanking primers (ANK2: F-ACCCACAGGACTGACTGAGG, R-ATGACTCCCTGAGGCTTTGA; FREM3: F-GGGCTGATAGGCAATCTTCA, R-CGATGATGATGGA GGTGGAT; IL15: F-TTTTTAGCCCAGTTGCAAGG, R-AATCAGGCCCAAAACACAAG; INTU:F-CCAGTTCCCTTAGGAGCACA, R-GGACTCCAGGGAGGTTGTCT; CLN7: F-CCTCGATATGCCTAATTAACACTG, R–AGTGAGGCCCCATCTCAAA; PLK4: F-TTTTGGCATGGGAGTTTAGC, R-AGTCTGAGGTGTCGGGTCTG). Amplicons were treated with Exonuclease I and Shrimp Alkaline phosphatase (New England Biolabs, Inc.) and sequenced on an ABI 3730XL DNA Analyzer (LifeTech, Inc.) by the ONPRC Molecular Biology Core. Sequence traces were analyzed using A Plasmid Editor software.

### 2.4. Magnetic resonance imaging

MRI examinations were performed on 5 Japanese macaques homozygous for the *CLN7* gene defect and 6 healthy control Japanese macaques (ages 2.9–5.8). All MRI data were acquired on a whole-body Siemens 3 Tesla (T) MRI instrument (Erlangen) using a quadrature radiofrequency (RF) coil with inner diameter of 15 cm. Animals were initially sedated with Telazol, intubated and maintained on 1% isoflurane in 100% O_2_ and were continuously monitored by pulse oximetry, respiration, and end tidal CO_2_ levels during the study. Quantitative R_1_ (≡1/T_1_) mapping was performed with a multiple-in-version recovery experiment employing 3D T1-weighted magnetic prepared rapid acquisition gradient echo (MPRAGE) sequence (TR: 2500 ms; TE: 3.49 ms; FA: 8°; FOV 130 mm × 97.5 mm × 96 mm; matrix: 192×144×96; TI: 200, 900, 2000 ms, no inversion). Cerebral and cerebellar volumes were calculated by a combination of linear and nonlinear co-registration with FSL (Jenkinson et al., 2012; Woolrich et al., 2009) to an atlas created in-house (unpublished data). Masks of the cerebellum were manually corrected to ensure accuracy in all cases. T2-weighted Turbo Spin Echo (TSE) sequences were acquired using the following parameters: FOV 120 × 160 mm; matrix = 320 × 240; slice thickness = 1.0 mm; FA = 120°; TR = 9000 ms; TE = 96 ms; number of averages = 2.

### 2.5. Retinal imaging

#### 2.5.1. Fundus autofluorescence

This technique was used as an *in vivo* measure of the accumulation of autofluorescent lysosomal storage material. Quantitative measurements of *in vivo* retinal fundus autofluorescence elicited by blue light excitation were obtained with a Heidelberg Spectralis spectral domain ocular coherence tomography (OCT) system with short-wavelength (488 nm) excitation laser, as previously described (McGill et al., 2016). This method used a manual gain setting that provides accurate readings across a wide range of autofluorescence intensities. Animals were anesthetized with Telazol (3–5 mg/kg IM) or with ketamine (10 mg/kg IM) followed by intubation and maintenance with isoflurane vaporized in 100% oxygen. The pupils were initially dilated with 2–3 drops each of 1% tropicamide and 10% phenylephrine, and a speculum and custom contact lens were inserted. The fundus was first bleached with a minimum 20-s exposure to the blue laser followed by acquisition of an image centered on the fovea. Each collected image was an average of 100 scans to improve the signal/noise ratio. The average mean grey value (MGV) of the autofluorescence image was determined using Image J. Measurements were obtained for the *CLN7^−/−^* monkey at 3.5, 4.7, and 5.3 years of age, and compared with cross-sectional data for 47 unaffected Japanese macaques across the lifespan.

#### 2.5.2. Optical coherence tomography

The Spectralis OCT system was also used to acquire high-resolution spectral domain OCT images from both eyes of each animal. OCT scans were acquired over a 30 × 20-degree field with 61 b-scans centered on the macula. Each slice was the average of ~20 images. Spectralis segmentation software (Eye Explorer 1.9.10.0) was used to segment 11 retinal layers, with subsequent inspection and manual correction by a skilled observer. Average thickness of the inner retina (external limiting membrane to the inner limiting membrane) and outer retina (inner limiting membrane to Bruch's membrane) were measured over a central 1 mm ring, a 1–3 mm ring, and a 3–6 mm ring centered on the fovea in 12 young controls and in BD6 at 3.5, 4.7, and 5.3 years of age.

#### 2.5.3. Electroretinography

Central cone-driven retinal function was assessed noninvasively with the multifocal electroretinogram (mfERG) as previously described (Hood et al., 2012; Koilkonda et al., 2014), using VERIS Science stimulus generation and response analysis hardware and software (Electrodiagnostic Imaging, Inc). Anesthesia was induced with a combination of ketamine (10 mg/kg IM), xylazine (1 mg/kg IM) and atropine (0.04 mg/kg IM) and maintained with additional quarter to full doses of each agent as necessary. Pupils were dilated, a custom bipolar Burian-Allen contact lens electrode was placed on the cornea, and corneal lubrication was maintained with hypromellose eyedrops; a subdermal needle electrode in the back served as ground. The ERG electrodes were fitted with + 3 diopter lenses to focus at the stimulus distance of 40 cm. A dynamic stimulus display on a high-resolution monitor subtended the central 40 degrees of the visual field and consisted of 241 unsealed hexagons each changing luminance from dark (1 cd/m^2^) to bright (~200 cd/m^2^) according to its own pseudorandom m-sequence at 13.3 msec intervals. The animal was placed prone in front of the display with its head positioned so that the center of the stimulus fell on the fovea, as determined by reverse direct ophthalmoscopy and initial alignment recordings. Recordings (8 min acquisition time) were acquired from each eye. Recordings were obtained from BD6 at 5 years of age and from 5 age-matched unaffected Japanese macaques. Response amplitudes (scalar product) and latencies of the major response waveform peaks were plotted for the fovea (“ring 1”) and 8 concentric rings of increasing eccentricity.

#### 2.5.4. Retinal tissue autofluorescence

Eyes were immersion-fixed for 24 h in buffered 4% paraformaldehyde (PFA) and then dissected to remove the anterior chamber and vitreous. The isolated eyecups were immersed in 4% PFA for an additional hour, cryoprotected using increasing concentrations of sucrose in phosphate buffered saline until the tissue sank in 30% sucrose, and then embedded in optimal cutting temperature (OCT) compound. They were oriented in horizontal plane in the mold and frozen on a liquid nitrogen cooled iron plate. Frozen sections were cut at 14 um thickness using a Leica CM1850 cryostat. Unstained single cross-sections of retinal tissue from BD6 and from 2- and 26-year-old unaffected Japanese macaques were used for qualitative comparative analysis of retinal autofluorescence. Posterior retina was imaged at approximately the same horizontal location in each subject using a confocal laser-scanning microscope using 488 nm laser power and gain settings. A color-merged z-stack comprised of 1 um scans throughout the entire tissue thickness was collected and used for comparison.

### 2.6. Electrocardiogram analysis

Electrocardiograms were performed using a Schiller AT-2 Light electrocardiograph machine with 6 limb leads. Animals were under ketamine sedation during the exam and four electrodes with conducting gel were applied, one per limb. Print-outs were made at speeds of 25 mm/s and 50 mm/s as well as amplitudes of 10 mm/mV and 20 mm/ mV. Interpretations were performed by the clinical veterinarian.

### 2.7. Necropsy and tissue collection

Animals were sedated with ketamine, deeply anesthetized with sodium pentobarbital intravenously and exsanguinated *via* the abdominal aorta. The cephalic structures were perfused with saline *via* the common trunk, brachiocephalic and right carotid arteries with 1.5 L of ice cold 0.9% sterile saline. Complete necropsies were performed and tissues from several major organs including brain, spinal cord and heart were collected, weighed and processed for histological examination.

### 2.8. Histochemistry

Tissues were fixed in 10% neutral buffered formalin, embedded in paraffin, sectioned at 5 μm and stained with hematoxylin and eosin (Leica ST5010 Autostainer XL, Leica Biosystems Inc.). Additional histochemical techniques performed on sections of the brain included the periodic acid-Schiff (PAS) reaction, PAS reaction with diastase digestion, and Luxol fast blue method, as previously described (C, C. F. A. H., 2009). A histochemical staining kit (American Master Tech) was used for Sudan black B. This procedure was modified by increasing the immersion time in the modified Sudan black B stain from five minutes to one hour. Reactive astrocytes (GFAP, 1:2000, DAKO) and microglia (Iba1, 1:1000; WAKO) were visualized using a biotin-labeled antibody procedure. Following endogenous peroxidase inhibition and washes, tissues were blocked for 1 h in 5% donkey serum, and primary antibody incubations were carried out for 24 h at room temperature. Sections were incubated in donkey anti-rabbit biotinylated IgG secondary antibodies (1:200; Vector Laboratories) for 1 h at room temperature. For neurofilament (NF) and myelin basic protein (MBP) staining, paraffin sections were deparaffinized and blocked with 5% normal goat serum then incubated with mouse anti-MBP (1:1000, Covance) and rabbit anti-NF, (1:500, Millipore). Secondary antibodies used were goat-anti-mouse immunoglobulin G (IgG) conjugated to Alexa 488 and goat-anti-rabbit IgG conjugated to Alexa 546 (1:500, Molecular Probes). The sections were then counterstained with 4,6-diamidino-2-phenylindole dihydrochloride (DAPI, 1:5000, Molecular Probes). In all staining procedures, deletion of the primary antibody served as a control. Sections were mounted onto gelatin-coated slides and coverslipped with Cytoseal 60 (Thermo Scientific). Images were captured by using an Olympus BX51 light microscope and DP72 digital camera or using a Zeiss Axioskop 40 equipped with epifluorescence and an AxioCam MRc digial camera system, which captured both immunohistochemical fluorescence labeling and autofluoresence.

### 2.9. Electron microscopy

Samples were fixed in cold Karnovsky fixative (2% PFA, 2.5% Glut in 0.1 M NaCacodylate buffer pH 7.2) and prepared for microwave-assisted processing in a Pelco BioWave Microwave. In the BioWave, samples were rinsed in 0.1 M Na Cacodylate buffer; incubated in reduced osmium tetroxide (1.5% potassium ferrocyanide in 2% OsO4); rinsed in water and *en bloc* stained with aqueous 0.5% uranyl acetate. Following the uranyl acetate incubation, samples were dehydrated in an aqueous series of 50%, 75% and 95% acetone, followed by two exchanges in 100% acetone. Epon resin infiltration was facilitated by incubation in a 1:1 solution of 100% acetone: freshly-made Epon resin, followed by 4 exchanges in 100% freshly-made Epon. Samples were removed from the BioWave and transferred into embedding capsules (BEEM) filled with freshly-made Epon and cured at 60 °C for 36 h. Thin sections (70 nm) obtained from the block face were imaged at 80 kV on a FEI-Tecnai 12 system interfaced to a digital camera and associated software (Advanced Microscopy Techniques).

### 2.10. Statistical analysis

Statistical analyses were performed using GraphPad software by Prism. MRI volumetric data was analyzed using a non-linear regression analysis to assess changes in volume of whole cerebrum, cerebral grey matter, cerebral white matter, whole cerebellum, cerebellar grey matter and cerebellar white matter over time in five BD and six control animals. Best fit values (slopes) were plotted for each data set. Whole brain weight data collected at necropsy was analyzed using a two-tailed, unpaired *t*-test analysis in five BD and three control animals. In all analyses, a *p*-value of ≤0.05 was considered significant. MRI and brain weight data are plotted as Mean ± SEM. All retinal data is presented as box-and-whisker plots showing the median, interquartile range, and highest and lowest values for each dataset.

### 2.11. Figure preparation

Figures were prepared using Adobe Photoshop, Prism GraphPad and Microsoft Powerpoint software.

### 2.12. Data availability

The datasets generated during and/or analyzed during the current study are available from the corresponding author on reasonable request.

## 3. Results

### 3.1. Clinical observations of progressive motor decline in a cohort of Japanese macaques at the ONPRC

Five Japanese macaque clinical cases, designated BD1, BD2, BD3, BD5 and BD6 presented at the ONPRC between 4 and 5 years of age with a relatively homogeneous and progressive constellation of neurologic symptoms including incoordination, hindlimb weakness, head tilt, intention tremor, ataxia, hypermetria and impaired balance (Table 1). Accordingly, affected animals displayed difficulty in ambulating, climbing, and jumping, and struggled with tasks requiring fine motor skills such as manipulating small objects. Aberrant motor behavior was first identified in free-ranging Japanese macaques housed in a 1-acre outdoor corral at the ONPRC. Once motor dysfunction was observed, animals were brought indoors to either larger group housing pens or individual home cages to maintain their safety, as well as for more detailed clinical observations. BD4 was taken to necropsy at 1.5 years of age and therefore we do not have detailed longitudinal behavioral observations for this animal. BD6 was the most well-characterized animal, presenting at age four with incoordination, intention tremor, a head tilt and ataxia. She also displayed hypermetria and impaired balance, evidenced by slips and falls when she misjudged the distance to the target during jumps onto elevated platforms or overreached in her efforts to grab for toys and food rewards. By age five, BD6 showed impaired balance during routine home cage ambulation, a delayed startle response and showed fine motor skll regression, with difficulty retrieving food rewards such as apples and peanuts out of recessed wells affixed to her home cage. Nearing the humane endpoint of disease manifestation, her movements became bradykinetic and home cage ambulation was slowed with frequent foot slips and misplacements. All affected animals reached a humane endpoint by age six.

BD7 was evaluated at age three and no major neurological deficits were found, to date, aside from slowed treat retrieval with each forelimb. BD8, BD9 and BD10 are currently 0.5–2.5 years of age, living in the outdoor Japanese macaque corral or in sheltered housing runs with disease progression being monitored regularly. Neither tonic-clonic nor myoclonic seizures, were observed in any of the Japanese macaque clinical cases. Supplemental Table 1 details the study participant demographics including animal ID, sex, genotype status, date of birth and the specific analyses for which they were used.

### 3.2. Genetic identification of the CLN7^−/−^ mutation

**Fig. 1.**
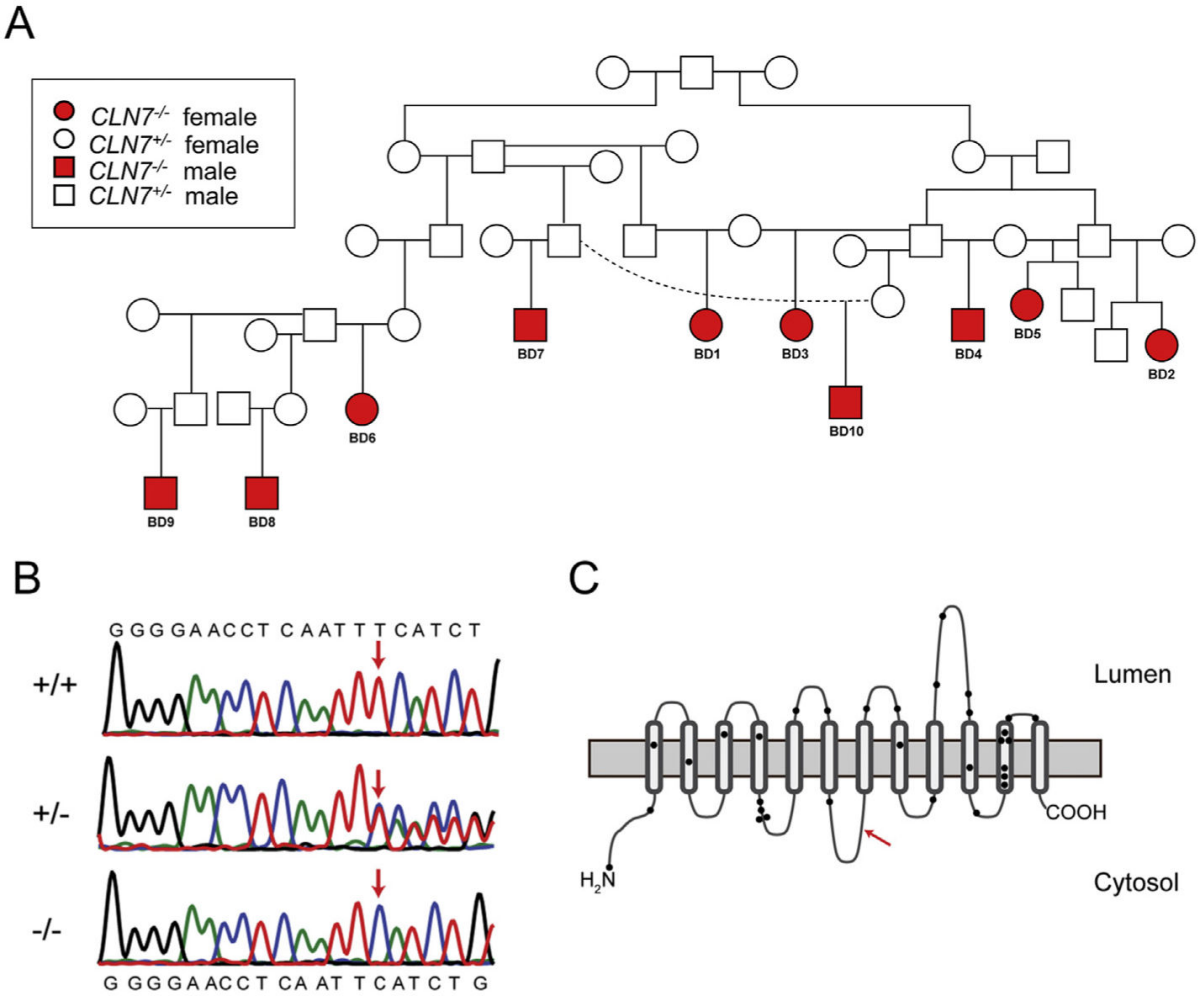
Genetic determination of the CLN7^−/−^ mutation in Japanese macaques. Ten *CLN7^−/−^* Japanese macaques (*Macaca fuscata*) have been identified at the ONPRC. Pedigree analysis indicates an autosomal recessive pattern of inheritance (A); affected *CLN7^−/−^* male and females are indicted with red circles and squares, respectively, while carrier *CLN7^+/−^* male and female kin are indicated by white circles and squares, respectively. Whole exome sequencing on *n* = 3 unaffected, n = 3 carrier and n = 3 affected animals identified a single base pair deletion in exon 8 of the *CLN7* gene, and 8 missense variants within flanking genes on Chromosome 4 in affected animals. DNA sequencing traces from unaffected *CLN7^+/+^*, heterozygous *CLN7^+/−^* carriers and affected homozygous *CLN7^−/−^* animals demonstrate the location of the single base pair deletion at position 769 in exon 8 on Chromosome 4, as indicated by the red arrows (B). The *CLN7* coding sequence is shown in the reverse direction relative to the sequence read and reference genome. The deletion at position 769 (CLN7c.769delA) confers a translational frameshift mutation. A schematic of the CLN7 protein with 12 transmembrane spanning domains is shown in (C), and the red arrow shows the position of the identified CLN7p.Ile257LeufsTer36 mutation in cytoplasmic domain 4, predicting to result in a truncation of the protein and loss of transmembrane domains 7–12. Black circles represent approximate positions of 31 reported missense and frameshift *CLN7* mutations in humans (Kousi et al., 2012). (For interpretation of the references to colour in this figure legend, the reader is referred to the web version of this article.)

Pedigree analysis of the affected macaques (Fig. 1A, represented as red circles and squares) and unaffected kin (represented as white circles and squares) suggested an autosomal recessive pattern of disease inheritance. Whole exome sequencing of three affected, three obligate carrier and three unaffected, unrelated animals produced 1,138,402,541 reads enriched for gene coding regions (Supplemental Table 2). In the absence of a Japanese macaque reference genome, reads were mapped to the closely related rhesus macaque reference genome, MacaM. In total, our variant analysis identified 11,445,775 variants. Of these, 702,329 variants were homozygous for the alternate allele in all sequenced individuals, likely indicating nucleotide positions that differentiate *Mucaca fuscata* from the *Macaca mulatta* genomes. Segregation analysis identified a single haplotype block of 177 variants located on chromosome 4 as associated with affected status of the 9 individuals sequenced (Supplemental Table 3; 106,658,369–146,307,114 bp). Within the haplotype block, 158/177 of the variants are within non-coding sequences. The 20 variants located within coding regions identified 8 predicted missense mutations and one single base frameshift mutation within 8 linked genes. Review of additional exon, whole genome and Sanger sequence data obtained from 73 NCL unaffected Japanese macaques, narrowed the associated nonsynonymous variants to one frameshift and 2 missense mutations (located within CLN7, *INTU, PLK4*, Supplemental Table 4). The single base deletion in *CLN7* in exon 8 (c.769delA; p.Ile257LeufsTer36) is predicted to terminate translation prematurely, resulting in the loss of transmembrane domains 7–12 in the encoded lysosomal transmembrane protein CLN7 (Fig. 1B, C Supplemental Table 4). Black circles depicted on the CLN7 protein schematic in Fig. 1C approximate the locations of the 31 human *CLN7* missense or non-sense mutations identified to date (Kousi et al., 2012). Using a Taqman-based genotyping assay that we developed, we confirmed homozygous frameshift alleles in all five affected individuals (BD1-BD5), and heterozygote alleles in all eight obligate carriers. Additional screening of the colony identified five more homozygous *CLN7^−/−^* Japanese macaques (termed BD6-BD10, Supplemental Table 1), all of whom were at or under the age of three and prior to the onset of overt behavioral decline. Further genotype screening of the ONPRC Japanese macaque colony identified a total of 59 heterozygote *CLN7^+/−^* carriers and 189 *CLN7^+/+^* macaques that do not carry the c.769delA variant. Consistent with the *CLN7* gene mutation being causative of a fatal disease, no homozygous mutant adult Japanese macaques over six years of age were identified.

### 3.3. Severe cerebral and cerebellar atrophy in CLN7 macaques

**Fig. 2.**
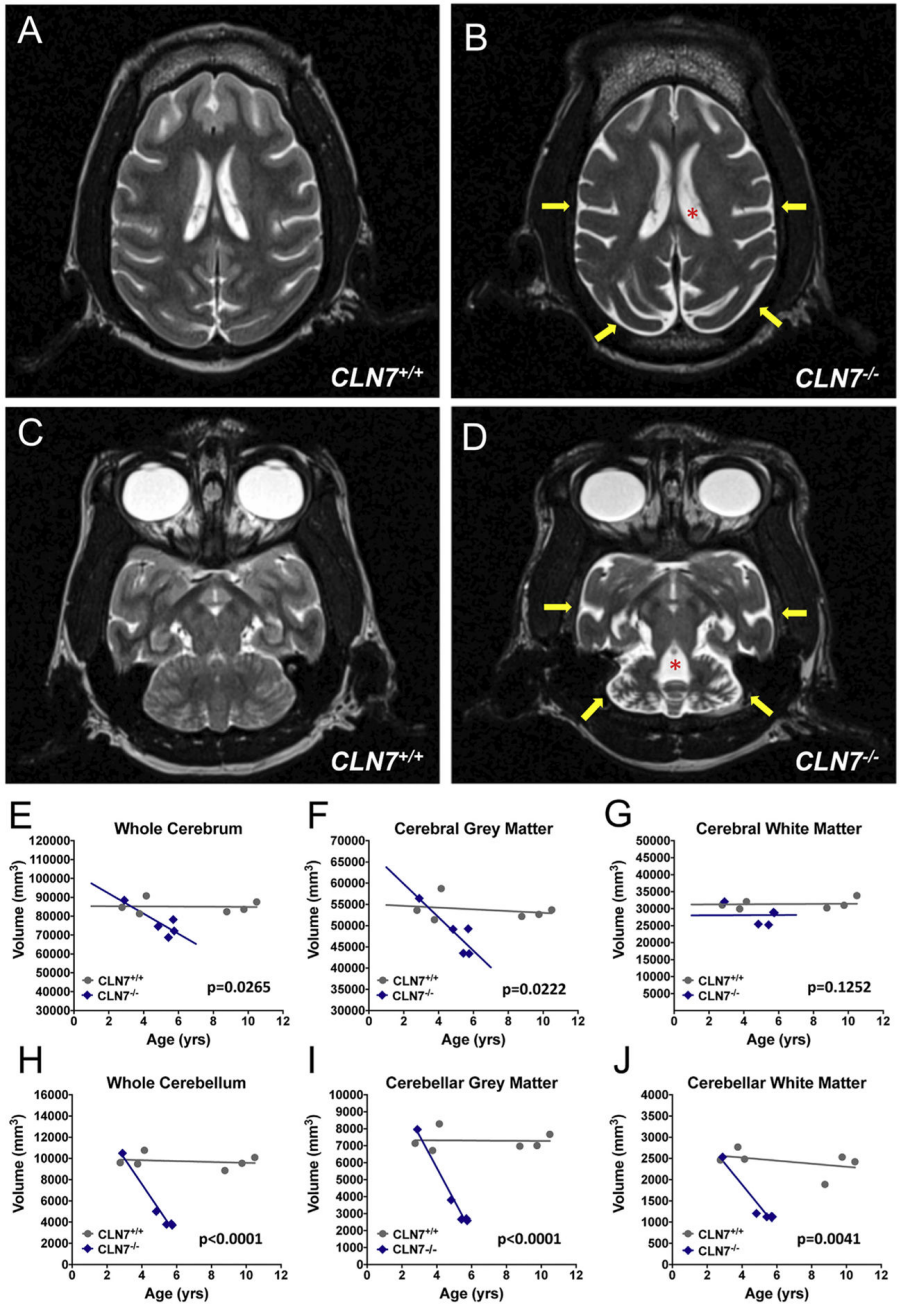
MRI analysis demonstrates significant *CLN7^−/−^* associated atrophy of the cerebrum and cerebellum. T2-weighted turbo spin echo scans of affected *CLN7^−/−^* mutant macaques indicate a dramatic reduction in both cerebral and cerebellar volumes (B, D) compared to *CLN7^+/+^* normal controls (A, C). Atrophy of affected macaque brains was associated with a gross enlargement of the ventricular system (indicated by red asterisks in B, D), with the fourth ventricle- adjacent to the cerebellum- being particularly affected (D). Increased cerebral spinal fluid (CSF) was observed surrounding the degenerating cerebral and cerebellar cortices in affected *CLN7^−/−^* animals compared to controls (indicated by yellow arrows in B, D). Volumetric analysis performed on quantitative R1 maps of *CLN7^−/−^* mutants (*n* = 5) and *CLN7^+/+^* controls (*n* = 6) demonstrate a progressive and significant mutation-associated reduction in whole cerebrum volume (*p* = .0265), largely driven by a reduction in the volume of cerebral grey matter (*p* = .0222), compared to controls (*E*-G). Atrophy of the cerebellum was particularly severe, wherein *CLN7^−/−^* BD macaques displayed a progressive reduction in whole cerebellar volume (*p* < 0.0001) compared to controls, driven by reductions in both cerebellar grey (*p* < .0001) and white matter (*p* = .0041) volumes (H-J). Differences between groups were evaluated using non-linear regression analyses, with *p* < .05 considered significant in all cases. (For interpretation of the references to colour in this figure legend, the reader is referred to the web version of this article.)

To investigate potential brain imaging correlates of behavioral decline in affected animals, we performed MRI examinations in 5 female *CLN7^−/−^* macaques, ages ranging from 2.9–5.8 years, and 6 healthy female controls ages ranging from 2.8–10.5 years using a Siemens Trio TIM 3.0 T MRI instrument located at the ONPRC. Axial T2-weighted Turbo Spin Echo (TSE) images revealed a substantial neurodegenerative process in *CLN7^−/−^* macaques compared to controls (Fig. 2A–D), evidenced by widespread atrophy throughout the brain, with prominent shrinkage in the forebrain (Fig. 2B) and cerebellum (Fig. 2D) compared to controls. A corresponding enlargement of the anterior and temporal horns of the lateral ventricle, as well as the fourth ventricle, were seen in *CLN7^−/−^* macaques compared to controls (indicated by asterisks in Fig. 2B, D). Additionally, *CLN7^−/−^* macaque brain scans demonstrated a subdural accumulation of cerebral spinal fluid (CSF) surrounding the forebrain and cerebellum compared to scans from the control cohort (indicated by yellow arrows in Fig. 2B, D).

Cerebral and cerebellar volumes (whole, grey matter and white matter) were calculated by a combination of linear and nonlinear coregistration of 3D T1-weighted magnetic prepared rapid acquisition gradient echo (MPRAGE) sequences to a macaque brain atlas created in-house. Cerebral volumes reported here are inclusive of the entire cerebrum (cortex, underlying white matter and sub-cortical structures) and do not include the cerebellum. Cerebellar volumes reported here include a small portion of the cerebellar peduncles but are primarily confined to the cerebellar lobes. Using non-linear regression analyses to compare the change in brain volume by age in each group, we found a significant reduction in both whole cerebrum (Fig. 2E, F = 7.84 (1,7), *p* = .0265) and cerebral grey matter (Fig. 2F, F = 8.555 (1,7), *p* = .0222) volumes over time across all *CLN7^−/−^* animals compared to controls (best fit values/slopes are plotted for each data set). No significant differences were seen between groups in cerebral white matter volume (Fig. 2G, F = 3.031 (1,7), *p* = .1252). We also observed a significant reduction in whole cerebellar (Fig. 2H, F = 74.58 (1,7), *p* < .0001), cerebellar grey (Fig. 2I, F = 66.03 (1,7), p < .0001) and cerebellar white matter (Fig. 2J, F = 17.48 (1,7), *p* = .0041) volumes with increasing age in *CLN7^−/−^* macaques compared to controls. Y-intercept, slope and standard error values for each statistical measurement are found in Supplemental Table 5. In both cerebral and cerebellar analyses, reduction in grey matter was more severe than changes in white matter.

### 3.4. Retinal pathology in macaques expressing the CLN7^−/−^ mutation

**Fig. 3.**
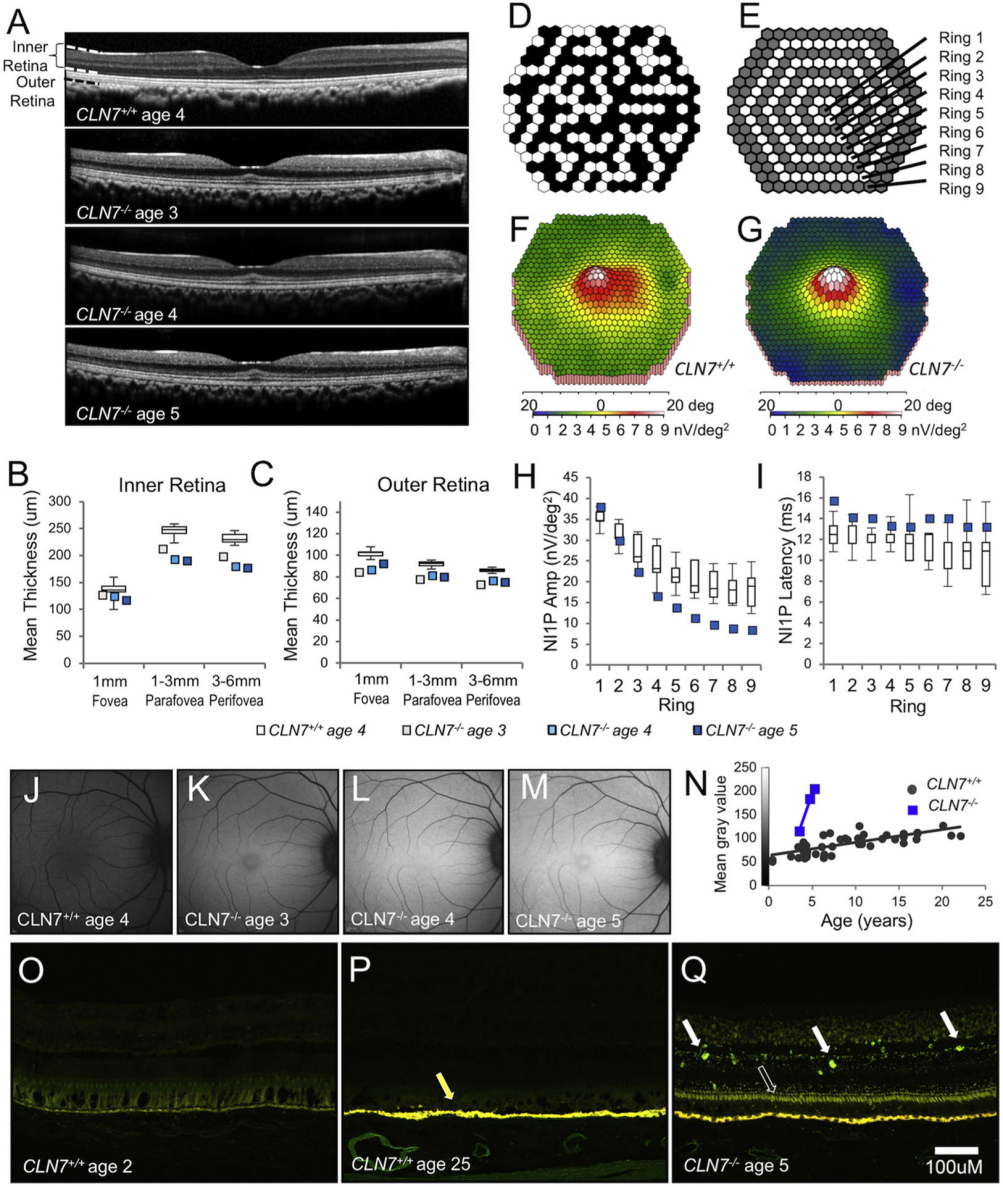
Figure *CLN7^−/−^* mutation is associated with structural and functional retinal abnormalities. OCT images illustrating retinal thinning in BD6 at ages 3.5, 4.7 and 5.3 compared to a 4-year-old *CLN7^+/+^* control (A). Analysis of these images showed a reduction in the thickness of both inner (B) and outer (C) retina layers in BD6 at each age compared to a cohort of controls (*n* = 12, data shown as box-and-whisker plot of median, interquartile range, and highest and lowest values). Values for BD6 were below the range of control values except in the foveal inner retina. (D) A single static frame of the mfERG stimulus with 241 hexagons subdivided into 9 rings (E) for further analysis. In comparison to the control (F), the mfERG false-colour map of response amplitudes shows a clear reduction in the outer rings but foveal sparing in BD6 at 5 years of age (G). In BD6, amplitudes were below the range of 5 age-matched controls (values shown as box-and-whisker plots) in rings 4–9 (H) and response latencies were increased, being above the control range in rings 1, 3, 6 and 7 (I). *In vivo* qFAF images indicate the accumulation of storage material in BD6 from ages 3.5, 4.7 and 5.3, compared to a 4-year-old *CLN7^+/+^* control (J-M). Autofluorescence intensity (mean grey values) in BD6 is shown in comparison to *CLN7^+/+^* controls across several ages (N). Retinal tissue sections from 2-year-old (O) and 25-year-old (P) *CLN7^+/+^* normal controls and 5-year-old BD6 (Q), show accumulation of autofluorescent storage material in BD6 throughout the photoreceptor inner segment layer (open arrow) and punctate deposits in the inner retinal layers (filled white arrows) compared to young or old controls. Yellow autofluorescence in the retinal pigment epithelium of the 25-year-old control (yellow arrow in P), is due to the typical accumulation of lipofuscin in older eyes. Scale bar in Q applies to frames O-Q. OCT-optical coherence tomography, mfERG-multifocal electroretinogram, qFAF-quantitative fundus autofluorescence. (For interpretation of the references to colour in this figure legend, the reader is referred to the web version of this article.)

Progressive loss of vision due to retinal degeneration is an early symptom of all NCLs, including the CLN7 form. To address whether the *CLN7^−/−^* mutation confers retinal degeneration in the BD macaques, we performed longitudinal optical coherence tomography (OCT) and quantified the thickness of retinal layers in the fovea (central 1 mm), parafovea (1–3 mm eccentricity) and perifovea (3–6 mm eccentricity) in BD6 compared to age-matched *CLN7^+/+^* controls (Fig. 3A). At ages 3.5–5.3, BD6 showed a reduction in thickness of both the inner and outer retinal layers compared to age-matched controls. Thickness of the inner retinal layers (from the external limiting membrane to the inner limiting membrane) was below the range of the controls in the parafoveal and perifoveal regions at age 3.5 (a reduction of 13%) and age 5.3 (reduction of 22%) but showed minimal loss in the fovea (Fig. 3B). In the outer retinal layers (from Bruch's membrane to the external limiting membrane), the foveal, parafoveal and perifoveal regions all exhibited thickness below the normal range, with values 10–17% below controls (Fig. 3C).

We next used multifocal electroretinography to examine the electrophysiological function of the macular cone photoreceptor system in BD6 at 5 years of age and in 5 age-matched controls. This method provides a map of the retinal response to luminance changes in an array of 241 hexagons subtending the central 40° of the retina (Fig. 3D–G). The resulting map of response amplitudes for each hexagon was grouped into 9 concentric rings (Fig. 3E). In concordance with the OCT data, BD6 showed spared function in the center of the fovea (rings 1 and 2) but severe loss of function in the outer rings (Fig. 3F, G). Response amplitudes in BD6 were below the control range in rings 4–9, with a reduction of over 50% in rings 6–9 (Fig. 3H); response latencies were increased, rising above the normal range in rings 1, 3, 6 and 7 (Fig. 3I).

In addition to impaired retinal function, BD6 displayed accumulation of autofluorescent storage material throughout the retina as measured by *in vivo* quantitative fundus autofluorescence (qFAF) (Fig. 3J–M). The intensity (mean grey value, MGV) of fundus autofluorescence in unaffected control animals averaged 73.0 ± 3.0 (SEM, *n* = 13) at 3 to 5 years of age and increased to 100.5 ± 5.5 by age 10–12 (*n* = 7) and 102.4 ± 7.2 at ages 20 and over (*n* = 5) (Fig. 3J, N). In contrast, BD6 had a MGV of 115 at age 3.5 (Fig. 3K, N) which increased to 184 by age 4.7 (Fig. 3L, N) and 206 by age 5.3 (Fig. 3M, N), representing increases of 57%, 152%, and 182%, respectively, over 3 to 5-year-old controls. These results were confirmed in the retinal tissue of BD6 at age 5.8 years. Compared with unaffected young (2-year-old, Fig. 3O) and aged (25-year-old, Fig. 3P) Japanese macaques, the retinal tissue of BD6 at age 5.8 years showed a marked increase in autofluorescence in all layers of the retina (Fig. 3Q). Specifically, autofluorescent storage material was present in the entire inner segment layer of the photoreceptors and as punctate deposits throughout the inner retinal layers, whereas minimal to no autofluorescence was observed in these layers in controls.

### 3.5. Hallmark accumulation of lysosomal storage material in CLN7^−/−^ macaque CNS

**Fig. 4.**
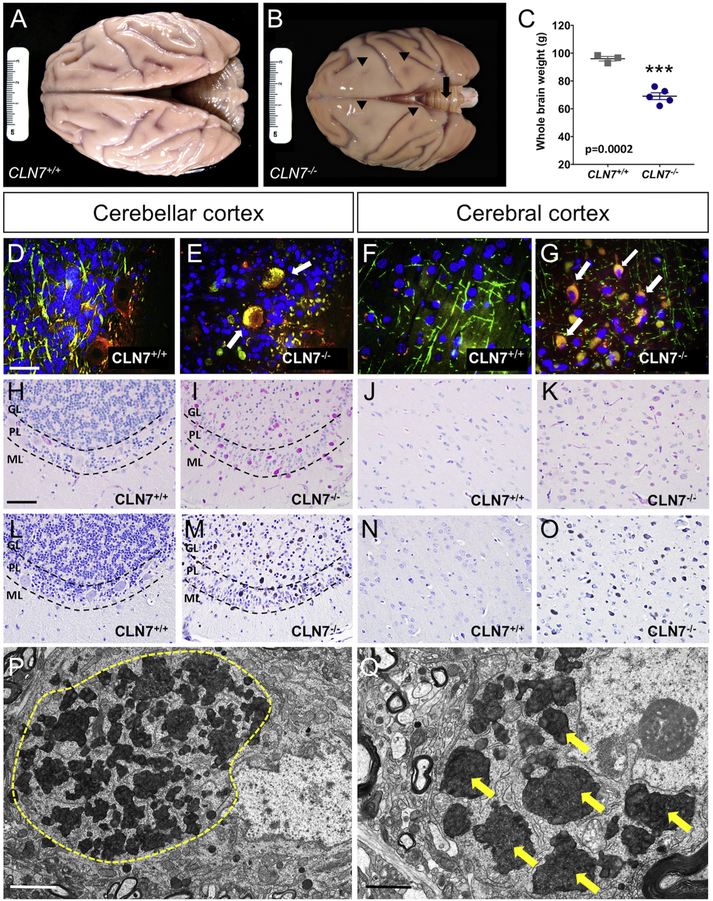
Hallmark accumulation of lysosomal storage material in *CLN7^−/−^* macaque CNS. Photographs of whole brains taken at necropsy from a *CLN7^+/+^* control (A) and a *CLN7^−/−^* mutant macaque (B), illustrate a dramatic reduction in whole brain size in affected animals. Shallow sulci in the frontal and parietal cortices of the affected animal shown here are indicated by black arrow heads (B) and atrophied cerebellum by a black arrow (B). Brain weight analysis from *CLN7^−/−^* macaques (*n* = 5) and age- and sex- matched CLN7^+/+^ controls (*n* = 3) and show a significant 28% reduction of whole brain weight in the mutant *CLN7^−/−^* macaques (C, *t*-test, *p*=.0002). Error bars indicate Mean ± SEM. Triple-labeled, cerebellar (E) and cerebral (G) cortical tissue sections for MBP (green), NF (red) and DAPI (blue) show marked accumulation of cytoplasmic, autofluorescent storage material in affected *CLN7^−/−^* macaque neurons (E, G, white arrows) compared to *CLN7^+/+^* controls (D, F). PAS (H–K) and SB (L–O) histochemistry illustrate the accumulation of glycoproteins and lipoproteins, respectively, in the cerebellar and cerebral cortex of affected *CLN7^−/−^* macaques (PAS-I, K; SB-M, O) compared to *CLN7^+/+^* controls (PAS-H, J; SB-L, N). Electron microscopy further confirmed cytoplasmic storage material characterized by the presence of granular osmiophilic deposits (GRODS) in neurons of both the cerebellar (P- a large cluster of GRODS is outlined in yellow) and cerebral (Q- small individual GRODS are noted by yellow arrows) cortex in affected *CLN7^−/−^* macaques. GL- granular cell layer, PL-Purkinje cell layer, ML-molecular cell layer. Scale bars in A, B = 3 cm, scale bar in D = 10 μm and applies to frames D–G, scale bar in H = 100 μm and applies to frames H–O, scale bar in *P* = 2μm, Scale bar in Q = 1 μm. (For interpretation of the references to colour in this figure legend, the reader is referred to the web version of this article.)

Brains were collected at necropsy after *CLN7^−/−^* animals reached a humane endpoint of their disease progression. Grossly, the brains from all *CLN7^−/−^* macaques were not only much smaller in size compared to those collected from *CLN7^+/+^* controls, but the BD brains were also discolored, characterized by a dark tan appearance (Fig. 4A, B). Atrophy of the *CLN7^−/−^* cerebrum and cerebellum was apparent and several of the cerebral and cerebellar sulci were very shallow, as indicated in Fig. 4B by the arrowheads and arrows, respectively. There was a significant 28% reduction in overall brain weight in *CLN7^−/−^* macaques compared to controls (Fig. 4C, *p* = .0002, *t* = 7.804, df = 6, unpaired *t*-test, Mean ± SEM).

The hallmark pathology in human NCL cases is the accumulation of highly autofluorescent, pigmented intracellular storage deposits. Initial examination of hematoxylin and eosin (H&E) stained sections of brains from affected animals revealed lightly pigmented grey-brown material that was highly autofluorescent, distending the cytoplasm of most neurons throughout the CNS. Unlike *CLN7^+/+^* controls, cerebellar and cerebral cortical tissue sections from *CLN7^−/−^* animals that were triple-labeled for myelin basic protein (MBP-green), neurofilament (NF-red) and 4′,6-diamidino-2-phenylindole (DAPI- blue) exhibited prominent accumulation of autofluorescent storage material that did not co-localize with DAPI, indicating an accumulation of storage material throughout the cytoplasm, with sparing of the nucleus (Fig. 4D–G). To further define the composition of the storage material, we stained the tissue from affected macaques and controls using Periodic acid-Schiff (PAS, Fig. 4H–K) and Sudan Black B (SB, Fig. 4L–O). The storage material in affected macaques appeared magenta with PAS staining and dusky black with SB staining, consistent with a composition of glycoproteins and lipoproteins respectively, which was not seen in controls. Intracellular storage material in both the cerebellum and cerebral cortex appeared turquoise with Luxol fast blue (LFB) staining, further confirming the presence of accumulated lipoproteins (not shown). Abundant storage material was found throughout most cerebellar and cerebral cortical layers of affected animals and, consistent with the MRI findings, the amount of storage material was greater in the cerebellum than in the cerebral cortex. Additionally, we found PAS-, SB- and LFB-positive storage material in sub-cortical brain regions of *CLN7^−/−^* animals that was not detected in controls including the dentate gyrus, CA regions of the hippocampus, thalamus, hypothalamus, caudate and putamen, albeit to a lesser degree compared to the cerebral and cerebellar cortices. Neurons of the spinal cord and dorsal root ganglia in affected animals were also PAS- and SB-positive compare to controls.

Electron microscopy has previously been used to categorize the ultrastructural characteristics of storage material profiles in tissue samples, such as peripheral blood lymphocytes and skin biopsies, collected from human NCL patients. Storage material inclusions are characterized as being membrane bound and having granular osmophilic deposits (GRODS), large curvilinear bodies, large vacuoles and/or fingerprint profiles, often corresponding to the specific forms of NCL (infantile, late infantile, juvenile or adult). We performed electron microscopy on cerebellar (Fig. 4P) and parietal cortex Fig. 4Q) tissue from *CLN7^−/−^* mutant animals, which revealed numerous large cytoplasmic clusters resembling GRODS, as well as a few examples of compact pigments containing curvilinear membrane profiles.

### 3.6. Neuronal loss, white matter degeneration and gliosis in cerebral cortex and cerebellum of CLN7^−/−^ macaques

**Fig. 5.**
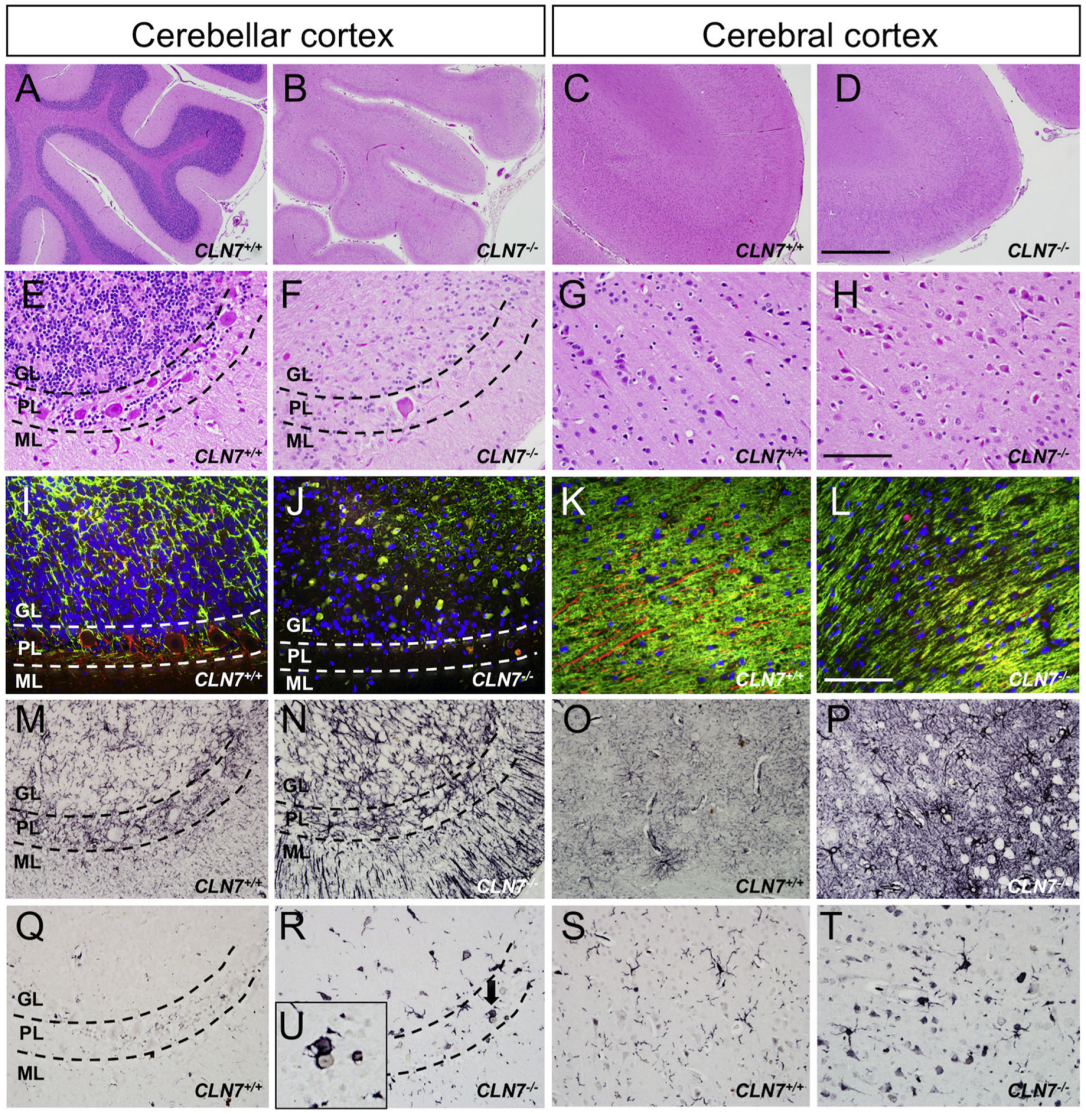
Neuropathological alterations in the *CLN7^−/−^* macaque brain. H&E stained sagittal cerebellar (A, B, E, F) and cerebral cortex (C, D, G, H) tissue sections from control and affected macaques showing atrophy in both brain regions, with the cerebellum more heavily affected. Triple-labeled, cerebellar (I, J) and cerebral cortex (K, L) tissue sections for MBP (green), NF (red) and DAPI (blue) show a loss of both neurons (loss of NF+ neurons) and white matter (loss of MBP+ axonal tracts) in affected *CLN7^−/−^* mutant macaques compared to controls. GFAP-(M–P) and Iba1-(Q–T) stained tissue sections from control and *CLN7^−/−^* animal cerebellar (M, N, Q, R) and cerebral (O, P, S, T) cortex highlight the robust astrogliosis and microgliosis, respectively, seen in the *CLN7^−/−^* mutant animals. Inset in U shows a reactive Iba1-positive microglial cell engulfing a degenerating cerebellar Purkinje cell in a *CLN7^−/−^* mutant animal. GL- granular cell layer, PL-Purkinje cell layer, ML-molecular cell layer. Scale bar in D = 500 μm and applies to frames A-D, scale bar in H = 100 μm and applies to frames *E*-H and M-T), scale bar in 1 = 50 μm and applies to frames I-L. (For interpretation of the references to colour in this figure legend, the reader is referred to the web version of this article.)

To further characterize *CLN7^−/−^* mutation-associated neuropathology, we performed a series of histological stains on sagittal-sectioned cerebellum and cerebral cortical tissue of affected macaques and age-matched controls to assess neuronal and white matter integrity as well as potential degeneration-associated gliosis. Hemotoxylin and Eosin (H&E) staining demonstrated a profound shrinkage of the *CLN7^−/−^* macaque cerebellar lobules compared to controls with a substantial loss of neurons in both the granule and Purkinje cell layers of the cerebellar cortex (Fig. 5A, B, E, F). A similar, yet less robust, atrophy of the cerebral cortex was also observed in H&*E*-stained brain sections from *CLN7^−/−^* animals compared to controls, with the example shown here from the parietal cortex (Fig. 5C, D, G, H), corroborating the cerebellar and cortical atrophy initially detected *via* MRI. Cerebellar tissue sections triple-labeled for MBP (green), neurofilament (red) and DAPI (blue) verified the loss of granular and Purkinje layer neurons (NF staining), and additionally illustrated a severe disintegration of myelin in cerebellar white matter (MBP staining) in affected animals compared to controls (Fig. 5I, J). A breakdown of NF-positive neuronal fibers and MBP-positive white matter tracts was also seen in the cerebrum of *CLN7^−/−^* animals Fig. 5K,L), but to a lesser degree compared to the cerebellum, again confirming the MRI analyses. To investigate a potential neuro-inflammatory response in affected brain regions we stained for two separate glial cell populations using antibodies against glial fibrillary acidic protein (GFAP) to evaluate astrocytes and against ionized calcium binding adaptor molecule 1 (Iba1) to assess microglia. Immunohistochemical staining demonstrated a very severe reactive astrocytic response throughout all layers of the *CLN7^−/−^* macaque cerebellum (Fig. 5M, N) and cerebral cortex (Fig. 5O, P), compared to *CLN7^+/+^* control animals. Similarly, we found an elevation in Iba-1 positive, microglia in both cerebellum and cortex of affected animals compared to controls (Fig. 5Q–T), noting several examples of activated microglia with extended processes as well as others with phagocytic processes wrapped around and actively engulfing degenerating neurons (Fig. 5U, inset). Together, these histological data support lysosomal dysfunction as an early and pervasive pathological event in this *CLN7* macaque model that is accompanied by robust neuronal and white matter degeneration and a severe inflammatory response.

### 3.7. Cardiac abnormalities and storage material accumulation

Because children with NCL often show cardiac dysfunction (Ostergaard et al., 2011), including altered electrocardiogram (ECG) traces, bradycardia, arrhythmia and storage accumulation, we investigated heart tissue from BD animals compared to controls. Postmortem, highly-autofluorescent storage material was present in cardiomyocytes of five *CLN7^−/−^* animals, which was not detected in controls. Additionally, we performed an ECG on an affected *CLN7^−/−^* animal and on a *CLN7^+/+^* age-, sex- and weight-matched normal control. Compared to the control animal, the CLN7^−/−^ animal showed a prolonged P wave width and PR interval, potentially indicating delayed conduction through the atrioventricular node (Supplemental Table 6). Furthermore, this animal showed a reduced heart rate (bradycardia) compared to the normal control. Each of these abnormal values also fell outside of the published normal range of Japanese macaque standard ECG measurements (Yamaoka et al., 2013).

## 4. Discussion

The family of NCLs remains a devastating group of pediatric diseases for which there are currently no cures. Having animal models available to the research community that recapitulate many of the same neuropathological and neurological manifestations seen in human patients is exceptionally valuable for studying disease processes, defining new biomarkers of disease progression and for testing promising new therapeutics. Here, we identify and characterize the first non-human primate model of any NCL identified to date. We discovered a small cluster of mutations, including a homozygous frame shift mutation in exon 8 of the *CLN7* gene on chromosome 4 in the Japanese macaque colony at the ONPRC, associated with progressive visual dysfunction and severe motor decline over a period of several years. The frame shift mutation discovered (c.769delA; p.Ile257LeufsTer36) is novel and not homologous to any of the previously documented *CLN7* mutations reported in human clinical cases (Kousi et al., 2012). The frameshift allele is predicted to result in a premature termination of the CLN7 protein and the formation of a truncated, non-functional lysosomal protein. It is also possible that truncated CLN7 is retained in the endoplasmic reticulum due to misfolding, also resulting in non-functionality of the protein. Additionally, we cannot formally rule out the possibility that the other two linked variants within the same haplotype block contribute to disease status.

The Japanese macaque CLN7^−/−^ disease phenotype that we characterized closely parallels the course of disease seen in children with the CLN7 form of Batten disease, including vision decline, tremor, hindlimb weakness, ataxia, incoordination, hypermetria, along with bradykinesia at later stages of disease (Table 2). We also saw intermittent head tilt in three animals, which has been seen in clinicals cases of cerebellar damage in both dogs and humans (Tamura et al., 2016; Baier et al., 2008) as well as in the recently described CLN7^−/−^ mutant dog cases (Faller et al., 2016). BD6 was the only animal of the original BD cohort (BD1-BD6) that was still alive at the time that the CLN7^−/−^ mutation was identified. Therefore, she had more numerous and frequent behavioral observations compared to the others. Through detailed cageside observations of BD6, we documented a profound loss of fine motor skills and a slowed startle response in this animal as her disease progressed, in addition to her other symptoms detailed above. Despite numerous common overlapping symptoms, one key symptom seen in human CLN7 disease that we did not observe in the affected *CLN7^−/−^* macaques is seizures, including tonic-clonic episodes or myoclonic jerks. Because seizures are often the first symptoms described to neurologists by parents of affected children with *CLN7* mutations, it is possible that we did not detect seizures in BD1-BD6 because they were living in a large outdoor corral among 200 other Japanese macaques. When less transient and more overt signs of motor impairment, such as incoordination and ataxia were apparent at age 4–5, they were readily detected by our clinical veterinarians. Therefore, it's plausible that these animals experienced seizures at earlier ages that went undetected. BD8-BD10, affected *CLN7^−/−^* macaques who are currently 0.5–2.5 years of age, are being monitored for potential seizure activity and future studies should include detailed experiments to systematically evaluate whether seizures are part of their disease-associated clinical repertoire.

The global cerebellar atrophy seen on T1- and T2-weighted MRI of CLN7^−/−^*Japanese* macaques, accumulation of autofluorescent storage material and severe depletion of cerebellar granular and Purkinje cell layers mirrors closely what is seen in human CLN7 patients, wherein the cerebellum is affected to a greater extent compared to the cerebral cortex. The fact that the cerebral cortex and cerebellum appear to take a more prominent pathological ‘hit’ compared to lesser affected regions such as the thalamus and basal ganglia, underscores the susceptibility of these brain regions to the mutation-associated pathological processes. A detailed investigation of the autofluorescent storage material detected in both the cerebral cortex and cerebellum confirmed that the hallmark accumulation of glyco- and lipoproteins with granular and curvilinear ultrastructure is similar to the composition of storage material observed in human CLN7 patients. We also detected autofluorescent storage material in cardiac tissue from five affected BD animals, which was not present in controls, suggesting that *CLN7^−/−^* associated pathological processes are also taking place outside of the CNS in this model, warranting further investigation in future identified mutant animals.

Because retinal pathology, including storage material accumulation, reduction in retinal layer thickness and altered electrophysiological response amplitudes can be detected *in vivo* using retinal imaging techniques such as qFAF, OCT and ERG, these modalities are invaluable as a first line of detection of NCL disease manifestation. In fact, human NCL cases are often first suspected by pediatric ophthalmologists during routine office visits upon detection of retinal dysfunction and visual abnormalities and are referred to neurologists for genetic testing. The availability of these sensitive *in vivo* retinal imaging techniques at the ONPRC allowed us to identify accumulation of fluorescent storage material and a reduction in retinal layer thickness in BD6 at three years of age, which progressively worsened over a three-year time course. However, BD4, who was euthanized at age 1.5, prior to reaching a humane endpoint of disease, already showed autofluorescent storage material in both retina and brain at the time of death, indicating that the lysosomal accumulation of cellular debris is an early pathological event in this non-human primate model that precedes both brain volumetric changes and motor decline.

A point of consideration is that all *CLN7^−/−^* affected macaques discussed here reached a humane endpoint and were euthanized by age 5.5–6 (aside from BD4), corresponding to a human age of roughly 16.5–18, primarily because they were at risk of injuring themselves due to severe incoordination and postural instability. Therefore, unlike the histopathological data from human cases which is from the fatal end-stage of the disease, the neuropathological changes seen *via* histology in our animals would be expected to be less severe compared to what is seen in the reported human CLN7 necropsy cases and can provide insights into earlier stages of disease pathogenesis that are not possible with material from human cases.

Despite there being a sizable number of genetically engineered mouse models available for many of the other NCLs (Shacka, 2012), only two CLN7 mouse models have been created to date (Brandenstein et al., 2016; Damme et al., 2014), likely owing to the later discovery of the *CLN7^−/−^* gene mutation in humans. A CLN7-lacZ gene trap hypomorphic mouse model was created first that recapitulates some of the neuropathological features of human CLN7 disease including accumulation of storage in CNS and periphery, along with reactive gliosis, however these mice show no evidence of motor decline nor premature death (Damme et al., 2014). A follow-up study by the same group describes an engineered knock-out mouse model *via* the removal of exon 2 of *CLN7*, leading to complete loss of CLN7 protein expression (Brandenstein et al., 2016). Interestingly, these mice show both phenotypic decline including hindlimb clasping, myoclonus and increased mortality, as well as a repertoire of CNS and cardiac deficits, highlighting the fact that a partial expression of CLN7 protein is sufficient to prohibit or delay clinical signs of disease (Brandenstein et al., 2016; Jankowiak et al., 2016). In contrast, the frameshift mutation (c.769delA) discovered in the CLN7 macaques predicts a premature stop codon and termination 36 amino acids after the frame shift (p.Ile257LeufsTer36). This termination is anticipated to result in a truncated and non-functional protein, which is in line with the behavioral and neuropathological signs of disease seen in the affected macaques. In addition to mice, small cohorts of Chihuahuas and one Chinese Crested Dog with naturally-occurring mutations in *CLN7* have recently been described (Ashwini et al., 2016; Faller et al., 2016; Guo et al., 2015). Affected Chihuahuas exhibited incoordination, head tilt and ataxia, similar to what we observed in *CLN7^−/−^* affected macaques. Additionally, MRI findings indicated generalized brain atrophy and enlarged lateral ventricles, and tissues at necropsy showed evidence of storage material accumulation in both CNS and heart, lack of frank cell loss but a reduced neuronal density in the cortex, granular and Purkinje cell loss in the cerebellum and retinal degeneration and gliosis, sharing many of the same pathological features as the *CLN7^−/−^* macaques described here. To our knowledge, *CLN7^−/−^* mutant canines have not been established as an animal model that is available to the BD community as a resource for research studies. See Table 2 for a comparison between each of the animals bearing *CLN7* mutations characterized to date, including our Japanese macaque model, and CLN7 disease in human patients.

We are currently expanding the numbers of *CLN7^−/−^* macaques at the ONPRC through a targeted breeding strategy and are collecting clinical data (MRI scans, retinal imaging and neurological assessments) and tissue samples to establish and maintain a database and tissue repository that can be utilized by investigators in the BD community. Given the low number of *CLN7^−/−^* animals models available to the BD research community, we are hopeful that the addition of this new macaque model will provide a valuable large animal model that can be used for further assessing disease pathology, developing biomarkers of disease progression and pre-clinical screening of promising therapeutics.

## Supplementary Material

1

## Figures and Tables

**Table 1:** Clinical characterization of *CLN7^−/−^* mutant macaque behavioral phenotypes.

*CLN7^−/−^* Animal ID	Clinical Phenotype Observed	Age at clinical observation
BD1	Incoordination, ataxia, head tilt, hindlimb weakness	5yrs
BD2	Incoordination, ataxia, head tilt, hindlimb weakness, impaired balance, hypermetria	5yrs
BD3	Incoordination, ataxia, hindlimb weakness, tremor, impaired balance, bradykinesia	5.5–5 yrs
BD5	Incoordination, ataxia, hindlimb weakness, tremor, impaired balance, bradykinesia	5–5.5 yrs
BD6	Incoordination, ataxia, head tilt, hindlimb weakness, tremor, impaired balance, hypermetria, impaired fine motor skills, impaired startle response, bradykinesia	4.5–5.5 yrs

*CLN7^−/−^* macaques were observed on multiple occasions by at least 2 independent veterinarians at the ONPRC at ages 4.5–5.5 years old. All *CLN7^−/−^* macaques displayed a progressive worsening of locomotor symptoms over the course of their disease.

**Table 2:** Comparison of novel macaque model of CLN7 disease to human CLN7 patients and other available models of the disease.

	*CLN7^−/−^* humans (Aiello et al., 2009; Kousi et al, 2009; Sharifi et al., 2010)	*CLN7^−/−^* macaques^#^	*CLN7^−/−^* dogs (Ashwini et al., 2016; Faller et al., 2016; Guo et al., 2015)	*CLN7^−/−^* mice (Brandenstein et al., 2016; Jankowiak et al., 2016)	*CLN7*^(tm1/tm1a)^ mice (Damme et al., 2014)
Neurological findings					
Ataxia	+	+	+	*NE*	−
Tremor	+	+	*NE*	+	−
Hypermetria	+	+	*NE*	*NE*	−
Head tilt	+	+	+	*NE*	−
Impaired balance	+	+	+	*NE*	−
Visual deficits	+	+	+	*NE*	*NE*
Seizures	+	−	+ /−	+	−
Cognitive deficits	+	*NE*	+	*NE*	*NE*
Mood disturbance	+	*NE*	+	*NE*	*NE*
Late-stage bradykinesia	+	+	*NE*	*NE*	−
Premature death	+	+	+	+	−
**Neuropathology findings**					
Accumulation of storage material in CNS	+	+	+	+	+
Regional brain atrophy	+	+	+	+	−
Enlarged ventricular system	+	+	+	+	−
Neuronal loss	+	+	+	+	−
White matter loss	+	+	*NE*	*NE*	*NE*
Astrocytosis	+	+	+	+	+
Microgliosis	+	+	+	+	+
Retinal degeneration	+	+	+	+	+
Impaired autophagy Peripheral pathology	+	*NE*	*NE*	+	*NE*
Accumulation of storage material in peripheral tissues	+	+	+	+	NE

+ denotes presence and - denotes absence of the neurological or neuropathological finding. NE (not evaluated) indicates that the animals were not measured for the specific phenotype.

# denotes findings from the current study.
