# Supplementary material for: Discovery of a CLN7 model of Batten disease in non-human primates
Source: Neurobiol Dis. Author manuscript; Available in PMC 2018 Nov 1. (PMC6200145; doi:10.1016/j.nbd.2018.07.013)
Supplement: 1 [file NIHMS990778-supplement-1.docx]

**SUPPLEMENTAL TABLES**

**Supplemental Table 1. Study participant information**

| Animal  ID | Sex | Genotype | Date of birth | | Data analysis inclusion |
| --- | --- | --- | --- | --- | --- |
| BD1 | Female | *CLN7 ^-/-^* | | 4/15/05 | Sequencing, MRI, behavior, histology |
| BD2 | Female | *CLN7 ^-/-^* | | 3/1/08 | Sequencing, MRI, behavior, histology |
| BD3 | Female | *CLN7 ^-/-^* | | 10/1/08 | Sequencing, MRI, behavior, histology |
| BD4 | Male | *CLN7 ^-/-^* | | 3/1/09 | Sequencing, MRI* |
| BD5 | Female | *CLN7 ^-/-^* | | 2/1/10 | Sequencing, MRI, behavior, histology |
| BD6 | Female | *CLN7 ^-/-^* | | 5/13/11 | Sequencing, MRI, mfERG, OCT,  behavior, histology, ECG |
| BD7 | Male | *CLN7 ^-/-^* | | 5/3/14 | Sequencing, behavior |
| BD8 | Male | *CLN7 ^-/-^* | | 7/10/15 | Sequencing |
| BD9 | Male | *CLN7 ^-/-^* | | 8/17/15 | Sequencing |
| BD10 | Male | *CLN7 ^-/-^* | | 6/29/17 | Sequencing |
| CTL1 | Female | *CLN7^+/+^* | | 3/15/06 | Sequencing, histology |
| CTL2 | Female | *CLN7 ^+/+^* | | 3/1/09 | Sequencing, MRI, histology |
| CTL3 | Male | *CLN7 ^+/+^* | | 4/15/07 | Sequencing, MRI |
| CTL4 | Male | *CLN7 ^+/+^* | | 2/1/09 | Sequencing, MRI |
| CTL5 | Male | *CLN7 ^+/+^* | | 3/1/09 | Sequencing, MRI |
| CTL6 | Male | *CLN7 ^+/+^* | | 2/1/09 | Sequencing, MRI |
| CTL7 | Female | *CLN7 ^+/+^* | | 6/1/04 | Sequencing, MRI |
| CTL8 | Female | *CLN7 ^+/+^* | | 10/1/04 | Sequencing, MRI |
| CTL9 | Male | *CLN7 ^+/+^* | | 6/26/02 | Sequencing, MRI, mfERG |
| CTL10 | Female | *CLN7 ^+/+^* | | 6/1/05 | Sequencing, MRI, mfERG |
| CTL11 | Male | *CLN7 ^+/+^* | | 4/1/03 | Sequencing, mfERG |
| CTL12 | Female | *CLN7 ^+/+^* | | 4/15/07 | Sequencing, mfERG |
| CTL13 | Male | *CLN7 ^+/+^* | | 2/15/06 | Sequencing, mfERG |
| CTL14 | Female | *CLN7 ^+/+^* | | 5/24/12 | Sequencing, OCT, FAF |
| CTL15 | Female | *CLN7 ^+/+^* | | 8/01/11 | Sequencing, OCT, FAF |
| CTL16 | Male | *CLN7 ^+/+^* | | 7/15/11 | Sequencing, OCT, FAF |
| CTL17 | Female | *CLN7 ^+/+^* | | 5/01/09 | Sequencing, OCT, FAF |
| CTL18 | Female | *CLN7 ^+/+^* | | 5/28/10 | Sequencing, OCT, FAF |
| CTL19 | Male | *CLN7 ^+/+^* | | 4/28/10 | Sequencing, OCT, FAF |
| CTL20 | Female | *CLN7 ^+/+^* | | 2/01/10 | Sequencing, OCT, FAF |
| CTL21 | Male | *CLN7 ^+/+^* | | 2/01/10 | Sequencing, OCT, FAF |
| CTL22 | Female | *CLN7 ^+/+^* | | 7/01/10 | Sequencing, OCT, FAF |
| CTL23 | Female | *CLN7 ^+/+^* | | 7/02/10 | Sequencing, OCT, FAF, ECG |
| CTL24 | Male | *CLN7 ^+/-^* | | 7/28/10 | Sequencing, OCT, FAF |
| CTL25 | Female | *CLN7 ^+/-^* | | 6/29/10 | Sequencing, OCT, FAF |
| CTL26 | Male | *CLN7 ^+/-^* | | 3/01/10 | Sequencing, OCT, FAF |
| CTL27 | Female | *CLN7 ^+/-^* | | 2/01/09 | Sequencing, FAF |

**Supplemental Table 1. Study participant information.** *animal was euthanized prior to overt development of a motor phenotype, BD-Batten Disease, CTL-Control, MRI- magnetic resonance imaging, mfERG- multifocal electroretinography, OCT- optical coherence tomography, ECG- electrocardiogram, FAF-retinal fundus autofluorescence.

| Supplemental Table 2. Summary of identified gene variants | | | |
| --- | --- | --- | --- |
| Variant Class | | | **Number of Variants** |
| Total Passing Filters | | | |
|  | Total | | 11,445,775 |
|  | SNP | | 9,878,451 |
|  | Non-SNP | | 1,567,324 |
| Common Homozygous | | | |
|  | Total | | 702,329 |
|  | SNP | | 636,980 |
|  | Non-SNP | | 65,349 |
| Passing Segregation Analysis | | | 178 |
|  | Non-coding | |  |
|  |  | Intergenic | 46 |
|  |  | Intronic | 84 |
|  |  | 5'UTR | 6 |
|  |  | 3'UTR | 17 |
|  | Coding | |  |
|  |  | Synonymous | 16 |
|  |  | Missense | 8 |
|  |  | Frameshift | 1 |

| Supplemental Table 3. All variants passing segregation analysis | | | | | | |
| --- | --- | --- | --- | --- | --- | --- |
| # | **Chr** | **Location(bp)** | **Reference Allele** | **Variant Allele** | **Variant Class** | **Gene /**  **Flanking Genes** |
| 1 | 4 | 106658369 | C | T | Intergenic | PAPSS1-SGMS2 |
| 2 | 4 | 108721146 | C | T | Intergenic | LRIT3/EGF |
| 3 | 4 | 108754610 | C | G | 5'UTR | EGF |
| 4 | 4 | 108754652 | G | A | 5'UTR | EGF |
| 5 | 4 | 108803181 | G | A | Intronic | EGF |
| 6 | 4 | 108854694 | ATTT | A | 3'UTR | EGF |
| 7 | 4 | 108881681 | G | A | Intergenic | EGF/ELOVL6 |
| 8 | 4 | 109200321 | A | G | Intergenic | ELOVL6/ENPEP |
| 9 | 4 | 109214251 | C | T | Intergenic | ELOVL6/ENPEP |
| 10 | 4 | 109523899 | A | G | Intronic | PITX2 |
| 11 | 4 | 111268570 | C | T | Synonymous | ALPK1 |
| 12 | 4 | 111317641 | G | A | Missense | ALPK1 |
| 13 | 4 | 111389100 | GC | G | Intergenic | NEUROG2 |
| 14 | 4 | 112135087 | A | C | Intronic | ANK2 |
| 15 | 4 | 112152505 | G | A | Intronic | ANK2 |
| 16 | 4 | 112216685 | C | T | Intronic | ANK2 |
| 17 | 4 | 112217477 | G | GT | Intronic | ANK2 |
| 18 | 4 | 112231357 | G | A | Intronic | ANK2 |
| 19 | 4 | 112234690 | C | T | Synonymous | ANK2 |
| 20 | 4 | 112255982 | G | A | Missense | ANK2 |
| 21 | 4 | 112256084 | A | G | Missense | ANK2 |
| 22 | 4 | 112256178 | T | C | Synonymous | ANK2 |
| 23 | 4 | 112257675 | G | A | Synonymous | ANK2 |
| 24 | 4 | 112258734 | G | A | Synonymous | ANK2 |
| 25 | 4 | 112263822 | C | T | Synonymous | ANK2 |
| 26 | 4 | 112281897 | A | G | 3'UTR | ANK2 |
| 27 | 4 | 112662832 | G | T | Intronic | CAMK2D |
| 28 | 4 | 112822411 | AAGTT | A | 3'UTR | ARSJ |
| 29 | 4 | 112822808 | G | GT | 3'UTR | ARSJ |
| 30 | 4 | 112824919 | T | C | Intronic | ARSJ |
| 31 | 4 | 112977855 | G | A | Intergenic | ARSJ/UGT8 |
| 32 | 4 | 113884196 | A | G | 3'UTR | NDST4 |
| 33 | 4 | 113992602 | GT | G | Intronic | NDST4 |
| 34 | 4 | 115013729 | A | G | Intergenic | NDST4/TRAM1L1 |
| 35 | 4 | 117382984 | T | C | Intronic | NDST3 |
| 36 | 4 | 117383629 | G | C | Intronic | NDST3 |
| 37 | 4 | 117383696 | C | CTG | Intronic | NDST3 |
| 38 | 4 | 117383711 | C | T | Intronic | NDST3 |
| 39 | 4 | 117383967 | G | A | Intronic | NDST3 |
| 40 | 4 | 117384312 | G | T | Intronic | NDST3 |
| 41 | 4 | 117384515 | G | A | Intronic | NDST3 |
| 42 | 4 | 117472138 | C | G | 3'UTR | PRSS12 |
| 43 | 4 | 117472637 | A | G | 3'UTR | PRSS12 |
| 44 | 4 | 117491054 | G | C | Intronic | PRSS12 |
| 45 | 4 | 117506373 | C | T | Intronic | PRSS12 |
| 46 | 4 | 117510960 | G | A | Intronic | PRSS12 |
| 47 | 4 | 117545925 | C | A | Synonymous | PRSS12 |
| 48 | 4 | 117593459 | G | A | Intergenic | PRSS12/METTL14 |
| 49 | 4 | 118241444 | CAATTAATTCTT  ATTAATTAATAA  GAATTCTTATT | C | Intergenic | FABP2/PDE5A |
| 50 | 4 | 118336065 | A | T | Intronic | PDE5A |
| 51 | 4 | 119139679 | G | T | Intergenic | MAD2L1/PRDM5 |
| 52 | 4 | 119474203 | C | T | Intergenic | MAD2L1/PRDM5 |
| 53 | 4 | 119592168 | C | T | Intronic | PRDM5 |
| 54 | 4 | 119609108 | A | G | Intronic | PRDM5 |
| 55 | 4 | 119609110 | T | A | Intronic | PRDM5 |
| 56 | 4 | 119609118 | G | A | Intronic | PRDM5 |
| 57 | 4 | 119609211 | C | T | Intronic | PRDM5 |
| 58 | 4 | 119812232 | T | G | Intergenic | PRDM5/NDNF |
| 59 | 4 | 119814438 | C | T | Intronic | NDNF |
| 60 | 4 | 119847343 | A | T | Intronic | NDNF |
| 61 | 4 | 119922063 | C | A | Intronic | TNIP3 |
| 62 | 4 | 119927889 | T | A | Intronic | TNIP3 |
| 63 | 4 | 119932564 | G | GA | Intronic | TNIP3 |
| 64 | 4 | 119942966 | C | G | Intronic | TNIP3 |
| 65 | 4 | 119944184 | G | A | Intronic | TNIP3 |
| 66 | 4 | 119951238 | A | T | Intronic | TNIP3 |
| 67 | 4 | 119968272 | G | A | Intronic | TNIP3 |
| 68 | 4 | 120115596 | T | C | Intronic | QRFPR |
| 69 | 4 | 120156606 | G | A | Intronic | QRFPR |
| 70 | 4 | 120156761 | G | A | Intronic | QRFPR |
| 71 | 4 | 120486354 | G | A | Intronic | ANXA5 |
| 72 | 4 | 120497331 | T | C | Intronic | ANXA5 |
| 73 | 4 | 120500763 | G | T | Intronic | ANXA5 |
| 74 | 4 | 120638139 | G | A | Intronic | CCNA2 |
| 75 | 4 | 120640350 | A | G | Intronic | CCNA2 |
| 76 | 4 | 120643205 | G | A | Intergenic | CCNA2/BBS7 |
| 77 | 4 | 120727610 | A | T | Intronic | TRPC3 |
| 78 | 4 | 120752545 | G | T | Synonymous | TRPC3 |
| 79 | 4 | 120760109 | G | A | Synonymous | TRPC3 |
| 80 | 4 | 121069634 | A | G | Intronic | KIAA1109 |
| 81 | 4 | 121112795 | T | C | Synonymous | KIAA1109 |
| 82 | 4 | 121151227 | C | T | Intronic | KIAA1109 |
| 83 | 4 | 121658268 | G | A | 5'UTR | BBS12 |
| 84 | 4 | 121835771 | G | A | 3'UTR | FGF2 |
| 85 | 4 | 121836652 | C | A | 3'UTR | FGF2 |
| 86 | 4 | 121862270 | G | C | Intronic | NUDT6 |
| 87 | 4 | 121874064 | C | T | Intronic | SPATA5 |
| 88 | 4 | 122365976 | A | T | Intergenic | SPATA5/SPRY1 |
| 89 | 4 | 122675279 | G | A | Intergenic | SPRY1/ANKRD50 |
| 90 | 4 | 124665187 | C | T | Intergenic | FAT4/INTU |
| 91 | 4 | 125585215 | T | G | Intergenic | FAT4-INTU |
| 92 | 4 | 125803685 | A | T | Intergenic | FAT4-INTU |
| 93 | 4 | 126920147 | G | A | Intronic | INTU |
| 94 | 4 | 126922121 | G | A | Missense | INTU |
| 95 | 4 | 126994237 | G | A | Intronic | SLC25A31 |
| 96 | 4 | 126995824 | G | T | Intronic | SLC25A31 |
| 97 | 4 | 127027835 | G | A | Synonymous | HSPA4L |
| 98 | 4 | 127033703 | T | G | Intronic | HSPA4L |
| 99 | 4 | 127058206 | G | A | Intronic | HSPA4L |
| 100 | 4 | 127064369 | A | G | 3'UTR | HSPA4L |
| 101 | 4 | 127106964 | A | C | Intronic | PLK4 |
| 102 | 4 | 127110632 | C | T | Missense | PLK4 |
| 103 | 4 | 127115820 | T | G | Intronic | PLK4 |
| 104 | 4 | 127120218 | G | A | Intronic | PLK4 |
| 105 | 4 | 127143487 | CA | C | 3'UTR | MFSD8 |
| 106 | 4 | 127143489 | A | T | 3'UTR | MFSD8 |
| 107 | 4 | 127144687 | G | C | 3'UTR | MFSD8 |
| 108 | 4 | 127160758 | AT | A | Frameshift | MFSD8 |
| 109 | 4 | 127224378 | G | C | Intergenic | MFSD8/C4orf29 |
| 110 | 4 | 127295943 | CTTATA | C | Intergenic | C4orf29/LARP1B |
| 111 | 4 | 127350449 | A | G | Intronic | LARP1B |
| 112 | 4 | 127368198 | A | G | Intronic | LARP1B |
| 113 | 4 | 127433319 | T | C | Intronic | LARP1B |
| 114 | 4 | 127561328 | C | A | 5'UTR | PGRMC2 |
| 115 | 4 | 127676510 | G | A | Intergenic | PGRMC2/PHF17 |
| 116 | 4 | 127995371 | C | T | Intergenic | PGRMC2/PHF17 |
| 117 | 4 | 128214484 | G | A | Intronic | SCLT1 |
| 118 | 4 | 128270725 | G | A | Intronic | SCLT1 |
| 119 | 4 | 128376453 | A | G | Intronic | C4orf33 |
| 120 | 4 | 128377962 | G | A | Intergenic | C4orf33/PCDH10 |
| 121 | 4 | 128394811 | T | C | Intergenic | C4orf33/PCDH10 |
| 122 | 4 | 128394933 | C | A | Intergenic | C4orf33/PCDH10 |
| 123 | 4 | 128395683 | A | G | Intergenic | C4orf33/PCDH10 |
| 124 | 4 | 128395800 | C | T | Intergenic | C4orf33/PCDH10 |
| 125 | 4 | 128397052 | AG | A | Intergenic | C4orf33/PCDH10 |
| 126 | 4 | 128397832 | A | G | Intergenic | C4orf33/PCDH10 |
| 127 | 4 | 128397834 | C | T | Intergenic | C4orf33/PCDH10 |
| 128 | 4 | 129227300 | G | A | Intergenic | C4orf33/PCDH10 |
| 129 | 4 | 130580519 | T | C | Intergenic | C4orf33/PCDH10 |
| 130 | 4 | 132264761 | G | C | Intergenic | C4orf33/PCDH10 |
| 131 | 4 | 132634748 | C | T | Intergenic | C4orf33/PCDH10 |
| 132 | 4 | 133098416 | T | C | Intergenic | PCDH10/PCDH1B |
| 133 | 4 | 133459832 | C | CCAAAA | Intergenic | PCDH10/PCDH1B |
| 134 | 4 | 133818988 | A | T | 3'UTR | PCDH10/PCDH1B |
| 135 | 4 | 136318909 | G | A | Intergenic | PCDH10/PCDH1B |
| 136 | 4 | 138015377 | C | A | Intergenic | PCDH1B/SLC7A11 |
| 137 | 4 | 138069223 | G | A | Synonymous | SLC7A11 |
| 138 | 4 | 138942607 | C | T | 5'UTR | CCRN4L |
| 139 | 4 | 138971219 | G | A | Intronic | CCRN4L |
| 140 | 4 | 138972843 | T | G | Synonymous | CCRN4L |
| 141 | 4 | 138973746 | ATCATT | A | Intergenic | CCRN4L/ELF4 |
| 142 | 4 | 138989372 | A | G | intronic | ELF2 |
| 143 | 4 | 139608040 | C | A | intronic | MGST2 |
| 144 | 4 | 139818997 | ATGGTAG | A | intronic | MAML3 |
| 145 | 4 | 140325353 | C | A | Intergenic | MAML3/SCOC |
| 146 | 4 | 140524584 | G | T | 3'UTR | ELMOD2 |
| 147 | 4 | 140716615 | G | A | intronic | TBC1D9 |
| 148 | 4 | 141056347 | C | A | Intronic | RNF150 |
| 149 | 4 | 141196521 | T | C | Intronic | ZNF330 |
| 150 | 4 | 141203796 | T | TTGGTTACA | Intronic | ZNF330 |
| 151 | 4 | 141203919 | C | T | Synonymous | ZNF330 |
| 152 | 4 | 141208733 | T | C | 3'UTR | ZNF330 |
| 153 | 4 | 141712413 | A | T | Missense | IL15 |
| 154 | 4 | 142003357 | G | C | Intergenic | IL15/INPP4B |
| 155 | 4 | 142003440 | A | C | Intergenic | IL15/INPP4B |
| 156 | 4 | 142015139 | A | G | Intronic | INPP4B |
| 157 | 4 | 142064232 | A | G | Intronic | INPP4B |
| 158 | 4 | 142067792 | T | C | Intronic | INPP4B |
| 159 | 4 | 142099422 | G | A | Intronic | INPP4B |
| 160 | 4 | 142099664 | C | G | Synonymous | INPP4B |
| 161 | 4 | 142318447 | C | T | Intronic | INPP4B |
| 162 | 4 | 143409996 | A | G | Intronic | GAB1 |
| 163 | 4 | 143410259 | C | T | Intronic | GAB1 |
| 164 | 4 | 143503893 | T | C | 5'UTR | SMARCA5 |
| 165 | 4 | 143540717 | A | AT | Intronic | SMARCA5 |
| 166 | 4 | 143687633 | C | T | Missense | FREM3 |
| 167 | 4 | 144902029 | CTTACA | C | Intronic | ABCE1 |
| 168 | 4 | 144902206 | T | A | Synonymous | ABCE1 |
| 169 | 4 | 144902603 | T | C | Intronic | ABCE1 |
| 170 | 4 | 144910309 | T | C | 3'UTR | ABCE1 |
| 171 | 4 | 144920883 | T | C | Intronic | OTUD4 |
| 172 | 4 | 144925930 | GA | GAA,G | Intronic | OTUD4 |
| 173 | 4 | 144947137 | T | TTA | Intronic | OTUD4 |
| 174 | 4 | 144954932 | A | G | Intronic | OTUD4 |
| 175 | 4 | 145384087 | A | C | Intergenic | SMAD1/MMAA |
| 176 | 4 | 145418815 | A | G | Missense | MMAA |
| 177 | 4 | 146307114 | T | TTCTTTG | Intergenic | SLC10A7/OU4F2 |
| 178 | 9 | 107006604 | G | A | Intergenic | WDR5/RXRA |

**Supplemental Table 4. Co-segregating coding variants associated with NCL affected status**

| Chr. | Location(bp) | Reference | Variant | Genes | cDNA Δ | AA Δ |
| --- | --- | --- | --- | --- | --- | --- |
| 4 | 126922121 | G | A | INTU | c.1282G>A | p.Val428Ile |
| 4 | 127110632 | C | T | PLK4 | c.1361C>T | p.Ser454Phe |
| 4 | 127160758 | AT | A | CLN7 | c.769delA | p.Ile257LeufsTer36 |

**Supplemental Table 5. MRI volumetric statistical data**

| Non-linear  regression analyses | Whole cerebrum | Cerebral grey matter | Cerebral white matter | Whole cerebellum | Cerebellar grey matter | Cerebellar white matter |
| --- | --- | --- | --- | --- | --- | --- |
| *CLN7^-/-^* BD mutant  Best fit  Y intercept | 102,716 | 67,692 | 35,024 | 17,263 | 13,363 | 3,900 |
| *CLN7^-/-^* BD mutant  Best Fit  Slope | -5,342 | -3,933 | -1,409 | -2,419 | -1,915 | -503.2 |
| *CLN7^-/-^* BD mutant  Standard Error  Y intercept | 10,005 | 6,365 | 5,550 | 1,068 | 706.1 | 395.8 |
| *CLN7^-/-^* BD mutant  Standard Error  Slope | 1,988 | 1,265 | 1,103 | 212.2 | 140.3 | 78.65 |
| *CLN7*^+/+^ Control  Best fit  Y intercept | 85,361 | 55,071 | 30,290 | 10,004 | 7,340 | 2,664 |
| *CLN7*^+/+^ Control  Best Fit  Slope | -44.29 | -205.2 | 160.9 | -41.59 | -5.697 | -35.89 |
| *CLN7*^+/+^ Control  Standard Error  Y intercept | 3,771 | 2,686 | 1,412 | 671.8 | 616.5 | 281.5 |
| *CLN7*^+/+^ Control  Standard Error  Slope | 515.4 | 367.1 | 193 | 91.81 | 84.26 | 38.48 |
| *F (DFn, DFd), p value* | 7.844 (1,7), p=0.0265 | 8.555 (1,7), p =0.0222 | 3.031 (1,7), p=0.1252 | 74.58 (1,7), p<.0001 | 66.03 (1,7), p<0.0001 | 17.48 (1,7), p=0041 |

**Supplemental Table 6. Electrocardiogram abnormalities in *CLN7^-/-^* affected macaque**

| Electrocardiogram values | *CLN7^+/+^* Control | *CLN7*^-/-^ BD macaque | Normal range* |
| --- | --- | --- | --- |
| Heart Rate (bpm) | 165 | **123** | 138 - 180 |
| P width (msec) | 48 | **80** | 23 - 59 |
| QRS width (msec) | 40 | 50 | 35 - 51 |
| PR interval (msec) | 80 | **120** | 69 - 91 |
| QT interval (msec) | 240 | 240 | 196 - 230 |
| P wave amp (mV) | 0.15 | 0.06 | 0.06 - 0.2 |
| R wave amp (mV) | 0.7 | 1.2 | 0.87 - 1.63 |
| T wave amp (mV) | 0.15 | 0.09 | 0.21 - 0.45 |
| QRS axis (degrees) | 30 | 60 | 57 - 93 |

*The normal range of electrocardiogram values for an adult Japanese macaque^25^; bpm- beats per minute, msec- milliseconds, mV- milliVolts
